# Incidence of intussusception in Singaporean children aged less than 2 years: a hospital-based prospective study

**DOI:** 10.1186/1471-2431-13-161

**Published:** 2013-10-08

**Authors:** Kong Boo Phua, Bee-Wah Lee, Seng Hock Quak, Anette Jacobsen, Harvey Teo, Kumaran Vadivelu-Pechai, Kusuma Gopala, Yanfang Liu

**Affiliations:** 1 KK Women’s and Children’s Hospital, Singapore, Singapore; 2 Mount Elizabeth Hospital, Singapore, Singapore; 3 National University Hospital, Singapore, Singapore; 4 GlaxoSmithKline Vaccines, Singapore, Singapore; 5 GlaxoSmithKline Pharmaceuticals, Bangalore, India

**Keywords:** Intussusception, Singapore, Hospital-based, Surveillance, Rotavirus vaccine

## Abstract

**Background:**

Continuous surveillance for intussusception (IS) is important for monitoring the safety of second-generation rotavirus vaccines. The present study aimed to assess the incidence of IS in Singaporean children aged < 2 years.

**Methods:**

This was a prospective, hospital-based, multi-center surveillance conducted in seven hospitals - two public hospitals and five private medical centers between May 2002 and June 2010 in Singapore. Diagnosis of IS (definite, probable, possible, suspected) was based on the case definition developed by the Brighton Collaboration. Children < 2 years of age who were diagnosed with IS were enrolled in this study. Incidence of IS was calculated per 100,000 child-year with its 95% confidence interval.

**Results:**

Of the 178 children enrolled, 167 children with definite IS cases were considered for final analyses; 11 were excluded (six diagnosed as probable IS and four diagnosed as suspected IS; one child’s parents withdrew consent). Mean age of children with definite IS was 11.6 ± 6 months; 67.7% were males. The overall incidence of IS was 28.9 (95% CI: 23.0–34.8) and 26.1 (95% CI: 22.2–30.0) per 100,000 child-year in children < 1 year and < 2 years of age, respectively. The majority of IS cases (20 [12.0%]) were reported in children aged 6 months. Most children (98.2% [164/167]) recovered, two (1.2%) children recovered with sequelae and one (0.6%) child died of septic shock.

**Conclusions:**

The incidence of IS remained low and stable in Singaporean children aged < 2 years during the study period (May 2002 to June 2010).

**Trial registration:**

NCT01177839

## Background

Intussusception (IS) is one of the most frequent causes of abdominal surgical emergencies in young children [1]. It occurs when one segment of bowel invaginates into the distal bowel, resulting in venous congestion and bowel wall edema [2]. Early diagnosis of IS is done using ultrasonography and/or air/hydrostatic enema. Air/hydrostatic enema is also used for the treatment of IS, in addition to surgical reduction of IS [3]. IS cases are most commonly observed in young children within the first year of life and are rare in children aged < 6 weeks, older children and adults [2]. The causes of IS are unknown in most cases. However, the frequent association of IS with intestinal lymphoid hyperplasia suggests that infectious agents may play a role [1].

Rotaviruses are the most important cause of severe diarrhea in young children accounting for over 453,000 deaths annually, worldwide [4]. In a study by Robinson CG, et al., it was demonstrated that rotavirus infection resulted in the thickening of distal ileal wall and mesenteric lymphadenopathy, suggesting a plausible mechanism of rotavirus-induced IS [5]. However, previous reports indicate no association between IS and rotavirus infection [6-8].

A first generation rotavirus vaccine, *Rotashield*® (Rotashield, Wyeth, Philadelphia, PA, USA), was withdrawn from market in the United States of America in 1999 as post-licensure surveillance revealed that administration of *Rotashield*® was temporally associated with an increased risk of IS and hence was withdrawn from the market by the manufacturer [9,10]. This incident has a considerable impact on the development and usage of next generation rotavirus vaccines [11,12].

Live attenuated oral rotavirus vaccines namely *Rotarix*™ (RIX4414, GlaxoSmithKline, Belgium) was licensed in Singapore in November 2005 and *RotaTeq*® (RV5, Merck and Co., Inc., USA) was licensed in Singapore in July 2007 [13]. These vaccines did not demonstrate any association with IS in large-scale, pre-licensure clinical trials [14,15]. Post marketing surveillance conducted in Mexico [16] and Australia [17] revealed an increased risk of IS following the administration of first dose of both these vaccines. However, recent studies have indicated that the benefits of rotavirus vaccines outweigh the risks [18,19]. To assess the risk-benefit ratio of the rotavirus vaccines in Singapore, it is, therefore, essential to collect the baseline information on the incidence of IS.

This study aimed to determine the incidence of IS in Singaporean children aged < 2 years through a prospective hospital-based surveillance conducted between 2002 and 2010.

## Methods

### Study design and study population

This prospective, hospital-based, multi-centre surveillance study was conducted in two public (KK Women's and Children's Hospital, the National University Hospital) and five private hospitals (Mount Elizabeth Hospital, Gleneagles Hospital, Mount Alvenia Hospital, East Shore Hospital, Thomson Medical Centre) in Singapore between May 2002 and July 2010. These hospitals treated the majority of IS cases in Singapore. The KK Women’s and Children’s Hospital alone treated nearly 72% of IS cases in Singapore [20]. Since all children with IS cases in Singapore were admitted to hospitals, this study covered almost all reported IS cases.

All children aged < 2 years and admitted to the study hospitals with a diagnosis of IS–categorized as definite (ascertained by: radiograph, surgery or by post-mortem examination), probable, possible or suspected cases based on the criteria developed by the Brighton Collaboration Working Group (version dated January 30, 2002) [21], were enrolled.

Data on IS were obtained from the daily admission logs, computerized hospital admission records, emergency department records, surgical records and radiology logs that were reviewed by the study staff and also from the discharge case notes that were written by surgeon to confirm the IS case. These were then keyed into the hospital database under the International Classification of Diseases (ICD) code for IS. In addition, the emergency department pediatric surgeons at each study site were involved to ensure that all IS cases were captured. All departments that were responsible for the management of IS cases were advised to contact the study personnel for each case of IS to ensure that all cases were captured. Each site gave an outline of their methods of surveillance, and provided evidence that case finding and ascertainment was sufficiently sensitive.

Clinical signs and symptoms of IS and vaccination history at the time of admission to hospitals were recorded in the case report forms. Children were excluded from the study if their age at the time of enrollment was ≥ 2 years or if the children had an episode of definite IS confirmed radiographically or surgically before enrollment.

Written informed consent forms were collected from children’s parents/ guardians prior to enrollment. This study was approved by the Institutional Review Board of each study centre in Singapore: KK Women’s and Children’s Hospital IRB/EC (KK Women’s and Children’s Hospital), National Healthcare Group HQ Domain Specific Review Board (DSRB) (National University Hospital) and Parkway Independent Ethics Committee (Mt. Elizabeth Hospital, Gleneagles Hospital, East Shore Hospital, Thomson Medical Centre and Mt. Alvenia Hospital). The study was conducted as per the principles of Good Clinical Practice, International Guidelines for Ethical Review of Epidemiological Studies, local regulations in Singapore and the Declaration of Helsinki.

### Diagnostic procedures

Abdominal radiograph, abdominal ultrasound, abdominal computed tomography (CT), gas/ liquid contrast enema and surgery were used to diagnose IS. The number of children who underwent each of these diagnostic procedures was recorded. The outcome of hospital admission was collected.

### Microbiology

Available stool samples were collected from subjects with definite IS as a part of routine diagnostic procedure. Microbiological examination of these stool samples was performed to determine the presence of any microbial (bacteria such as *Escherichia coli*, *Campylobacter*, *Salmonella*, *Yersinia* and *Shigella*) and/or viral (namely rotavirus) pathogens. Microbial detection was done using stool culture and rotavirus was detected using commercial test kits (antigen detection, latex agglutination and immunochromatographic tests).

### Statistical analyses

The incidence of IS was calculated using the following formula:

$$\text{Annual}\;\text{Incidence}\;\text{of}\;\text{IS}=\frac{\text{Number}\;\text{of}\;\text{new}\;\text{IS}\;\text{cases}\;\text{reported}\;\text{in}\;a\;\text{specific}\;\text{year}}{\text{The}\;\text{total}\;\text{number}\;\text{of}\;\text{children}\;\text{living}\;\text{in}\;\mathrm{Singapore}\;\text{during}\;\text{the}\;\text{specific}\;\text{year}}\times 100,000$$

The base population included all children aged < 2 years living in Singapore during the specific year.

The incidence of IS among children aged < 1 year and < 2 years were calculated with their respective exact 95% confidence intervals (95% CI). Incidence of IS was expressed per 100,000 child-year.

The trend in incidence of IS hospitalizations from May 2002 to July 2010 was assessed. Further, seasonal variation in the occurrence of IS by month was also assessed for the entire study period.

## Results

### Demography

A total of 178 children were assessed for IS of which six were probable, four were suspected IS cases. The remaining 168 children were definite IS cases. Of these, one child’s parent withdrew consent after enrolment. A total of 167 children were thus enrolled and included in the final analyses.

The mean age of children included in the final analyses was 11.62 ± 6.22 months; 67.7% of enrolled children were males. Among these children, 17/167 and 1/167 children received RIX4414 and RV5, respectively. Following the administration of RIX4414, IS case was reported by 2/17 children seven days post-Dose 1 and 1/17 child between 7–31 days post-Dose 2. Children receiving RV5 did not report IS cases within 31 days following any dose.

**Figure 1:**
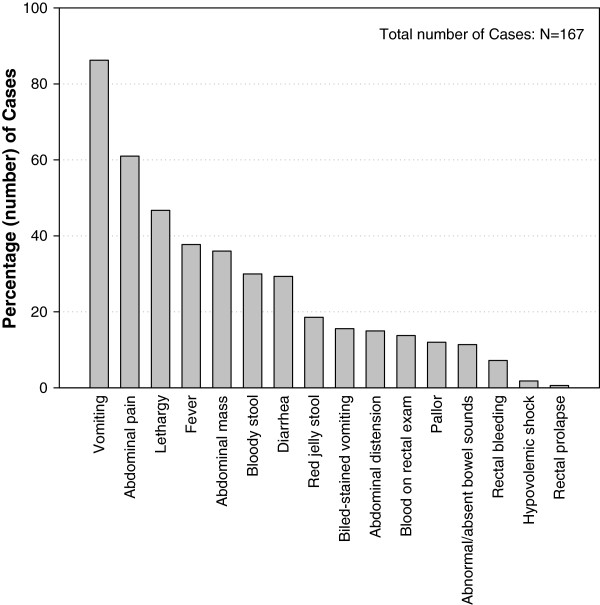
Clinical symptoms observed in children with definite IS case (Total number of cases N=167).

Vomiting (144/167 [86.7%]), lethargy (78/167 [65%]) and abdominal mass (60/167 [38%]) were the most commonly reported clinical symptoms among children hospitalized with definite IS cases (Figure 1).

### Incidence

**Figure 2:**
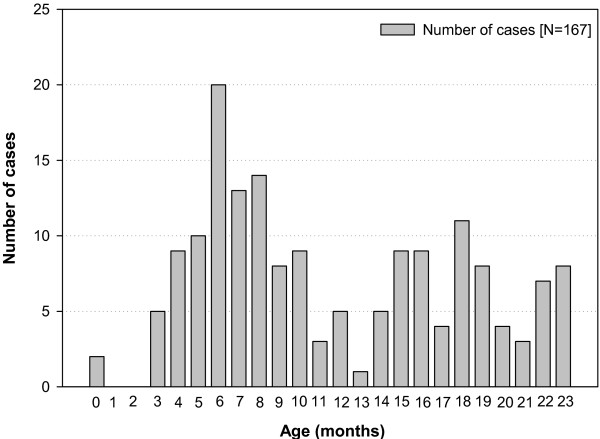
Distribution of IS cases by age (Total number of cases N=167).

**Figure 3:**
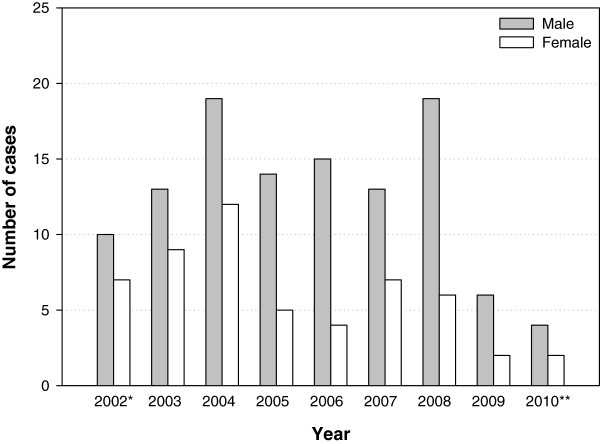
**Distribution of IS cases by gender (Total number of cases N=167).** *Data collected from May to December - 2002. **Data collected from January to June - 2010.

The overall incidence of IS observed in children aged < 1 year and < 2 years was 28.9 (95% CI: 23.0–34.8) and 26.1 (95% CI: 22.2–30.0) per 100,000 child-year, respectively. The annual incidence of IS during the entire study period is detailed in Table 1. The highest number of IS cases (20/167 [12.0%]) were reported in the age group of six months (Figure 2). The number of IS cases during the entire study period did not show any clear seasonal pattern (data not shown). The number of IS cases were predominantly higher in males than in females (Figure 3).

**Table 1:** **Incidence of IS among children** < **1 and** < **2 years of age** (**N**=**167**)

**Year**	**Incidence per 100**,**000 child**-**year**					
	**Children< 1 year**	**95% CI**		**Children < 2 years**	**95% CI**	
		**LL**	**UL**		**LL**	**UL**
2002*	44.5	19.3	69.7	31.5	16.6	46.5
2003	32.1	13.9	50.3	29.4	17.1	41.7
2004	35.0	16.0	54.0	43.0	28.1	57.9
2005	37.4	17.8	57.1	25.4	14.0	36.8
2006	28.8	11.8	45.8	24.9	13.7	36.1
2007	33.0	15.1	51.0	25.4	14.3	36.5
2008	27.6	11.3	43.9	31.4	19.1	43.7
2009	7.6	0.0	16.2	10.1	3.1	17.2
2010**	15.3	0.3	30.3	11.5	2.3	20.7

95% CI: exact 95% confidence interval; LL: lower limit; UL: upper limit.

Children < 2 years include children < 1 year and children aged 1–2 years.

*Data collected from May to December - 2002.

**Data collected from January to June - 2010.

### Diagnostic procedures

The majority of children (166/167 [99.4%]) underwent abdominal ultrasound; abdominal radiograph was performed on 96/167 (57.5%) children and gas/ liquid contrast enema was performed on 95/167 (56.9%) children. Furthermore, 33/167 (19.8%) children underwent surgery and bowel resection was performed in 18/167 (10.8%) children.

### Outcomes

Of the 167 children hospitalized with definite IS, 164 (98.2%) recovered completely, while two children (1.2%) who underwent surgery recovered with sequelae and one child died of septic shock.

One child who recovered with sequele had a successful reduction of IS by surgery but developed *E*. *coli* septicaemia and acute tubular necrosis which required dialysis. One child developed pneumoperitoneum following air enema. This child underwent laparotomy and hemicolectomy was carried out because of serosal split. However, this child recovered fully. The child who died during the study period had undergone air reduction and had recovered from IS. Despite having fully recovered from the IS episode, the child died a month later. Autopsy revealed necrotising enterocolitis, severe hyaline membrane disease, severe generalized lymphocytic depletion and haemosidrosis of the liver and spleen.

### Microbiology

A total of 53 (31.7%) stool samples were available from 167 children for microbiological analyses. Microbial examination revealed that three distinct samples were positive for *Salmonella* while *Escherichia coli* and *Campylobacter* were found in one distinct sample each. There were no mixed infections. Rotavirus was not isolated from any of the samples tested.

## Discussion

This prospective hospital-based surveillance spanning a period of eight years was the largest study aimed at providing recent estimates of IS incidence in Singapore. During the eight year surveillance period, the observed incidence of IS was low with an overall incidence estimated at 28.9 and 26.1 per 100,000 child-year in children < 1 and < 2 years of age, respectively. This is in line with the previously observed incidence of IS in Singapore, where the incidence ranged between 26.4 and 39.9 per 100,000 among children aged < 1 year and between 23.8 and 28.7 per 100,000 among children aged < 2 years, during 2005–2007 [13]. The IS incidence values observed in Singapore in the present study and the one conducted previously were lower than that observed in Taiwan (77.0 and 93.5 per 100,000 child-year in children < 1 and < 2 years of age, respectively) [22] and Germany (60.4 and 51.5 per 100,000 child-year in children < 1 and < 2 years of age, respectively) [23]. Although the exact reason for this low IS incidence is unknown, a study conducted by Tan N et al., [13] indicated that the baseline incidence of IS was lower in Singapore.

Furthermore, the present results indicate that during the eight year surveillance, the incidence of IS was lowest in 2009. Although the exact reason for the lower IS incidence rate observed in 2009 in the present study is unknown, natural fluctuation of IS cases might have caused this effect.

The present study demonstrated a higher incidence of IS in children aged < 1 year as compared to children aged < 2 years which is in accordance with a previously conducted study in Singapore [13]. A review of published literature between 1966 and 2001 by the World Health Organization on the worldwide IS incidence also showed that the incidence of IS was higher in children aged < 1 year than in children aged < 2 years [2]. Furthermore, the peak incidence of IS was observed between 4–8 months of age, with highest number of cases reported at six months of age. These results were also similar to the results observed in other studies conducted in Taiwan [22], Korea [24], Thailand [25], Australia [26] and also the United States where two-thirds of IS cases occur below the age of one year [27].

While the peak incidence of IS was observed at six months of age, a secondary peak was observed at 18 months of age, which was similar to previous findings in Taiwan and Australia [22,26]. However the exact reason for this peak in the incidence of IS at 18 months is not known.

Of the 53 available stool samples collected from definite cases of IS, none of the samples tested positive for rotavirus. These results indicate that, among the children with definite IS and from whom stool samples were collected, the cause of IS can be assumed to be non-rotavirus related.

There were several limitations which might affect the interpretation of results observed in this present study. The information regarding the application of different diagnostic procedures stepwise was not captured in this study. Therefore, it was not possible to determine the number of IS cases confirmed by a single diagnostic procedure. Since there was no distinction between diagnostic and therapeutic procedures, it was not possible to classify the therapeutic procedures explicitly. For instance, children who underwent surgery might have had another treatment measure prior to surgery and surgery was opted for only when the other treatment measures were unsuccessful. These limitations might have led to potential bias in reporting the diagnostic/ treatment procedures in Singapore. Lastly, stool samples were not actively collected as part of this study. Microbial examination was performed only on stool samples that were collected from children during routine clinical examination and analyzed. Furthermore, the microbial examination did not include specific tests for the detection of non-enteric viruses such as adenovirus.

## Conclusions

This study provides the baseline information on the incidence of IS which might aid in assessing the risk-benefit ratio of rotavirus vaccines in Singapore.

Incidence of IS remained stable and low during the eight year surveillance period from May 2002 to June 2010 in Singaporean children aged < 2 years. Children aged < 1 year were more frequently affected with IS than children < 2 years of age. The therapeutic procedures carried out at the study hospitals demonstrated successful reduction of IS in majority of children.

### Trademark statement

*Rotashield* is a registered trademark of Wyeth group of companies.

*Rotarix* is a trademark of GlaxoSmithKline group of companies.

*RotaTeq* is a registered trademark of Merck and Co., Inc. group of companies.

## Abbreviations

CI: Confidence interval; CT: Computed tomography; GSK: GlaxoSmithKline; IS: Intussusceptions.

## Competing interests

Phua Kong Boo: received money for travel related to the study in the past.

Lee Bee Wah: Has received consultancy fees, honorarium and money for travel related to the study and payment for lectures including speaker’s bureau.

Anette Jacobsen: No conflict of interest to declare.

Quak Seng Hock: Received support for travel expenses related to the study and also received travel, accommodation and meeting expenses unrelated to the study.

Harvey Teo: No conflict of interest to declare.

Yanfang Liu and Kumaran Vadivelu: Employees of GlaxoSmithKline group of companies and hold shares of GlaxoSmithKline.

Kusuma Gopala: Employee of GlaxoSmithKline group of companies.

## Authors’ contributions

PKB, LBW, QSH, AJ, HT, have provided input towards the design, conduct, review and interpretation of results from the study and critical review and approval of the manuscript, in addition to contribution towards subject enrollment. KV was involved during the study and provided input into the clinical study report and critically reviewed the content of the manuscript and approved it. KG was involved in the statistical analyses, interpretation, critical review and input towards the protocol, study result interpretation and critical review and approval of the manuscript. YL was involved in all the scientific aspects relating to the study design, input towards analyses and interpretation of the results, critical review and approval of the manuscript. All authors read and approved the final manuscript.

## Pre-publication history

The pre-publication history for this paper can be accessed here:

http://www.biomedcentral.com/1471-2431/13/161/prepub
